# Perfusion cardiovascular magnetic resonance and fractional flow reserve in patients with angiographic multi-vessel coronary artery disease

**DOI:** 10.1186/s12968-016-0263-0

**Published:** 2016-07-19

**Authors:** Shazia T. Hussain, Amedeo Chiribiri, Geraint Morton, Nuno Bettencourt, Andreas Schuster, Matthias Paul, Divaka Perera, Eike Nagel

**Affiliations:** Papworth Hospital NHS trust, Papworth Everard, Papworth Everard, Cambridgeshire UK; King’s College London BHF Centre of Excellence, NIHR Biomedical Research Centre and Welcome Trust and EPSRC Medical Engineering Centre at Guy’s and St. Thomas’ NHS Foundation Trust, Division of Imaging Sciences, The Rayne Institute, London, UK; Portsmouth Hospitals NHS trust, Portsmouth, UK; Centro Hospitalar de Vila Nova de Gaia/Espinho, EPE, Vila Nova de Gaia, Portugal; Department of Cardiology and Pulmonology and German Centre for Cardiovascular Research, Göttingen, Germany; Luzerner Kantonsspital, 6000 Luzern 16, Switzerland; King’s College London BHF Centre of Excellence, NIHR Biomedical Research Centre at Guy’s and St. Thomas’ NHS Foundation Trust, Cardiovascular Division, The Rayne Institute, London, UK; DZHK Centre for Cardiovascular Imaging, University Hospital Frankfurt/Main, Frankfurt/Main, Germany; Cardiology Department, Papworth Hospital, Papworth Everard, CB23 3RE UK

**Keywords:** Ischemia, Fractional flow reserve, Coronary artery disease, Perfusion CMR

## Abstract

**Background:**

Perfusion cardiovascular magnetic resonance (CMR) and fractional flow reserve (FFR) are emerging as the most accurate tools for the assessment of myocardial ischemia noninvasively or in the catheter laboratory. However, there is limited data comparing CMR and FFR in patients with multi-vessel disease. This study aims to evaluate the correlation between myocardial ischemia detected by CMR with FFR in patients with multivessel coronary disease at angiography.

**Methods and results:**

Forty-one patients (123 vascular territories) with angiographic 2- or 3-vessel coronary artery disease (visual stenosis >50 %) underwent high-resolution adenosine stress perfusion CMR at 1.5 T and FFR measurement. An FFR value of <0.75 was considered significant.

On a per patient basis, CMR and FFR detected identical ischemic territories in 19 patients (46 %) (mean number of territories 0.7+/−0.7 in both (*p* = 1.0)). On a per vessel basis, 89 out of 123 territories demonstrated concordance between the CMR and FFR results (72 %). In 34 % of the study population, CMR resulted in fewer ischemic territories than FFR; in 12 % CMR resulted in more ischemic territories than FFR. There was good concordance between the two methods to detect myocardial ischemia on a per-patient (k =0.658 95 % CI 0.383-0.933) level and moderate concordance on a per-vessel (k = 0.453 95 % CI 0.294–0.612) basis.

**Conclusions:**

There is good concordance between perfusion CMR and FFR for the identification of myocardial ischemia in patients with multi-vessel disease. However, some discrepancy remains and at this stage it is unclear whether CMR underestimates or FFR overestimates the number of ischemic segments in multi-vessel disease.

## Background

Revascularization of patients with stable coronary artery disease (CAD) should be guided by functional information rather than anatomy [1, 2]. A large body of evidence for the non-invasive assessment of ischemia is based on single photon emission computed tomography (SPECT), however, especially in the last decade, cardiovascular magnetic resonance (CMR) has shown advantages such as higher spatial resolution [3, 4] and potentially better diagnostic accuracy [5].

Guiding revascularization by fractional flow reserve (FFR) has demonstrated improved outcome in comparison to anatomy-guided strategies [6]. The accuracy of CMR and FFR for the detection of CAD has been well demonstrated and comparative studies have shown excellent diagnostic accuracy of perfusion CMR to detect functionally significant CAD identified by FFR [3, 7]. However, there are limited data on their comparability in defining ischemic segments in patients with multi-vessel disease. Detection of 3VD with non-invasive imaging can be challenging due to the effects of balanced ischemia leading to false-negative results in up to 20 % of cases [8]. A comparative accuracy study done by Chung et al. [9] compared SPECT and perfusion CMR in patients with angiographically proven three vessel disease and showed that CMR detected perfusion defects in all three vascular territories in 57 % of patients vs only 11 % with SPECT. Data comparing SPECT and FFR have also shown fewer ischemic territories with SPECT than FFR in this group [10]. The low spatial resolution of SPECT may also lead to underestimation of perfusion defects [11].

It is unknown, whether the use of a high-resolution perfusion technique such as CMR leads to improved concordance for the identification of ischemic segments in multi-vessel disease in comparison with FFR. The aim of this study was to compare the extent of myocardial ischemia based on CMR and FFR in patients with angiographically defined multi-vessel disease.

## Methods

The study was approved by the Kings College London (KCL) research ethics committee and all patients gave written informed consent to participate. Potential participants were identified after elective diagnostic coronary angiography and informed consent was obtained. A total of 41 patients with inclusion criteria of angina and stable 2- or 3-vessel disease designated on a visual basis by angiography (diameter stenosis >50 %) were recruited. All patients underwent FFR assessment during the subsequent PCI procedure and CMR (performed as part of the research protocol) which occurred prior (within 4 weeks) to the PCI procedure.

Exclusion criteria were contra-indications to CMR (i.e., claustrophobia, metallic implant, pacemaker), contra-indications to adenosine therapy, previous coronary artery bypass graft (CABG), left main stem disease, recent myocardial infarction (MI) within 6 months, unstable angina and left ventricular (LV) ejection fraction <30 %.

### CMR image acquisition

Data were acquired with a 1.5 T scanner (Achieva, Philips, Best, The Netherlands) using 32-channel coils. Examinations included high-resolution perfusion, cine and scar imaging. Perfusion imaging consisted of 3 short axis slices acquired every heartbeat covering 16 of the standard myocardial segments (apex excluded) [12] first during adenosine stress followed by a short axis cine imaging stack and then rest perfusion imaging. Imaging parameters for perfusion imaging: kt BLAST acceleration factor 5 SSFP sequence, shortest TE (range 1.35–1.54 ms), shortest TR (range 2.64–3.12 ms), 50° flip angle; 90° prepulse, 100 ms prepulse delay and typical acquired resolution 1.7 × 1.9 × 10 mm. 0.075 mmol of weight adjusted contrast agent (Gadobutrol/Gadovist, Bayer Healthcare, Germany) was injected at 4 ml/s by a power injector, followed by a 20 ml flush for stress imaging with adenosine infused according to a standard adenosine protocol (140 μg/kg/min for 3 min, if no response after 2 min increase to 170 μg/kg/min). There was a 10 min delay between stress and rest imaging. The cine images were completed with a set of long axis views. Late Gadolinium enhancement (LGE) images were acquired after 10 min (Gadovist 0.2 mmol/kg cumulative dose) using an inversion recovery sequence.

### CMR image analysis

Perfusion CMR images were analyzed by two experienced observers blinded to the angiographic data and clinical history (AC and SH). They reported all scans with consensus; any disagreement was arbitrated by a third reader (EN). The CMR images were also graded for quality on a grading system of 1 (poor), 2(moderate) and 3 (good).

A perfusion defect was defined as reduced contrast uptake at peak stress persisting for 5 consecutive heart beats but not present at rest. Corresponding late gadolinium enhanced images were reviewed side by side with the perfusion data and enhanced myocardium was disregarded for ischemia.

Designation of vascular territories was done according to AHA 16 segment classification [13] Segments 1, 2, 7, 8, 13, and 14, were assigned to the left anterior descending coronary artery (LAD). Segments 3, 4, 9, 10, and 15 were assigned to the right coronary artery (RCA). Segments 5, 6, 11, 12, and 16 were assigned to the left circumflex artery (CX). This analysis was performed without knowledge of angiographic variation as per clinical practice.

### Coronary angiography and FFR measurement

After obtaining arterial access, a standard Judkin’s technique was used to obtain angiographic views. Intracoronary pressure measurements were obtained in all vessels that showed a ≥50 % diameter stenosis, assessed angiographically, using a 0.014-inch intracoronary pressure wire (Volcano Therapeutics, San Diego, CA, USA, or Pressure-Wire Certus, St Jude Medical Systems AB, Uppsala, Sweden). FFR was calculated during hyperemia (intravenous adenosine infused at 140 micrograms kg/min for at least 90 s) as P_d_/P_a_, where P_d_ and P_a_ are distal coronary and aortic pressure respectively. In cases of serial stenoses or when there was diffuse disease, the pressure sensor was positioned beyond the most distal diseased segment and if the FFR indicated hemodynamically significant disease, this was ascribed to the most proximal lesion for the purpose of this analysis. A FFR of <0.75 was considered significant. Coronary occlusions or lesions of ≥99 % were defined as FFR positive. Arteries with angiographic plaque < 50 % diameter stenosis were defined as FFR negative.

### Statistical analysis

Data analysis was performed with SPSS version 20 (SPSS Inc., Chicago Illinois). Continuous variables were presented as mean ± standard deviation (SD). The k statistic values were derived to investigate per-patient and per-vessel concordance between FFR and CMR derived evidence for ischemia (a k statistic of +1 indicating perfect agreement, 0 indicating agreement as expected by chance, and −1 indicating complete disagreement). In groups where kappa statistics could not be performed (i.e., where the value in one group was constant) concordance was assessed by percentage agreement.

A subgroup analysis according to different FFR thresholds was also performed. Where possible the kappa statistic was used, otherwise percentage agreement was used.

## Results

All 41 patients (29 males, average age 62 ± 9 years) and 123 territories were included in the analysis Table 1 summarizes the clinical characteristics of the patients and the angiographic features. There was an adequate stress response during the CMR scan with a mean heart rate increase from 63 to 80 and an increase in rate pressure product from 8243 to 9889. 27 were good quality scans, 12 were moderate and 2 scans were of poor diagnostic quality Two patients had subendocardial scar, none had transmural scarring. Within the 123 arteries, there were 10 occluded vessels.

**Table 1:** Pt clinical characteristics and angiographic details

Parameter		Number or mean (+/− SD)
Age (years)		62 (9)
Sex (male)		30
Body Mass Index		27.8 (4.1)
CAD risk factors (%)		
	Diabetes	20.6
	Hypertension	60.0
	Smoking	14.3
	Hypercholesterolaemia	93.3
Previous PCI		18.5
Previous myocardial infarction		4.0
Drug therapy (%)		
Aspirin		81.5
Statin		80.0
B blocker		61.3
ACE I		29.6
Angiographic details		
Vessels with FFR >0.75		72
Vessels with FFR ≤0.75		51

Abbreviations: *ACEI* angiotensin converting enzyme, *FFR* fractional flow reserve, *PCI* percutaneous intervention

### Comparison of CMR and FFR

**Fig. 1:**
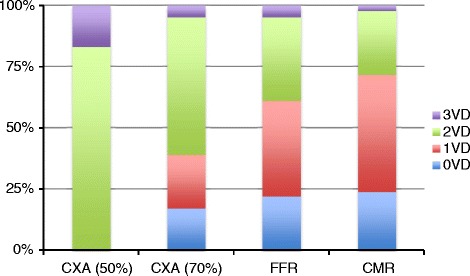
Respective proportion of number of vascular abnormalities as described by Coronary angiography (based on a angiographic cut off of 50 % and 70 % stenosis), CMR and FFR (CXA = coronary x-ray angiography, CMR = cardiovascular magnetic resonance, FFR = fractional flow reserve)

If an angiographic cut-off of 50 % stenosis was used to define patients with multivessel disease, 34 patients had 2VD and 7 pts had 3VD (See Fig. 1).

If an angiographic cut-off of 70 % stenosis was used 7 patients had 0 vessel disease (17 %), 9 patients 1- vessel disease (22 %), 23 patients 2- vessel disease (56 %) and 2 patient 3- vessel disease (4 %).

CMR demonstrated no perfusion defect in 10 patients (24 %), ischemia in one territory in 20 (49 %) patients, two territories in 11 patients (27 %) and 3 territories in one patient (2 %). All cases were read with consensus between two readers with only two cases requiring a third observer.

FFR results were negative in all vessels in 9 patients (22 %), positive in 1 vessel in 16 patients (39 %), in 2 vessels in 13 patients (32 %) and 3 vessels in 3 patients (7 %).

The mean number of territories identified per patient was 1.0 ± 0.8 by CMR and 1.2 ± 0.9 by FFR.

### Concordance between FFR and CMR

In 22 patients (54 %), there was complete agreement as to the number of territories of ischemia: mean number of territories 0.7 ± 0.7 for both (*p* = 1.0). Of these there was concordance in territories identified in 93 % of patients.

In 6 patients (15 %), CMR showed more ischemic territories than FFR, in 13 patients (32 %), CMR showed fewer ischemic territories than FFR (See Table 2).

**Table 2:** Concordance between CMR and FFR on a per patient basis according to number of significant FFR values and CMR perfusion defects

		FFR result			
		0	1	2	3
CMR result	0	7	2	0	1
	1	1	11	8	0
	2	1	3	4	2
	3	0	0	1	0

Abbreviations: *CMR* cardiovascular magnetic resonance, *FFR* fractional flow reserve

**Fig. 2:**
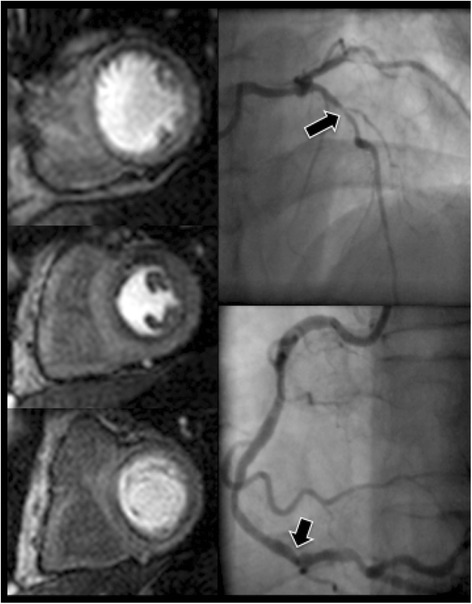
Case example of concordance between FFR value and CMR. Angiographic images and corresponding perfusion images of a patient with 2-vessel disease. The LAD has a proximal stenosis (FFR value 0.63) (see *arrow*) resulting in a perfusion defect in the anterior wall visible in the apical, mid and basal ventricular slice. The RCA has a distal stenosis (FFR value 0.62) (see *arrow*) resulting in a perfusion defect in the inferior wall visible in the basal and mid slice. (Abbreviations: CMR = cardiovascular magnetic resonance, FFR = fractional flow reserve, LAD = left anterior descending artery, RCA = right coronary artery)

**Fig. 3:**
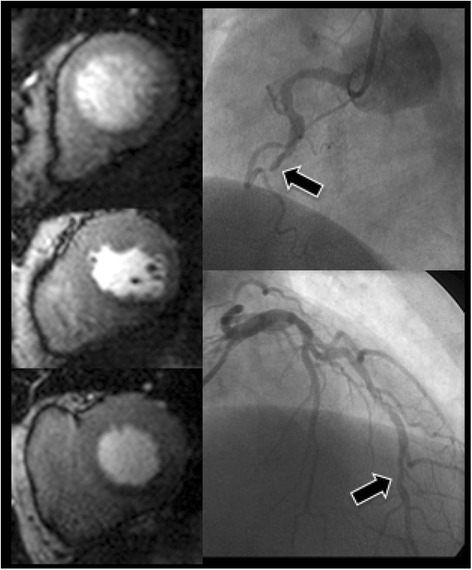
Case Example of discordance between the FFR value and CMR. Angiographic images and corresponding perfusion images of a patient with 2-vessel disease. The LAD has a distal stenosis (FFR value 0.7) (arrow) with no associated perfusion defect. The RCA has a proximally occluded artery (arrow) resulting in a perfusion defect in the inferior wall visible in all three slices. The combination of a distal lesion and a mildly positive FFR value in the LAD results in no demonstrable ischemia in the anterior wall. (Abbreviations: CMR = cardiovascular magnetic resonance, FFR = fractional flow reserve, LAD = left anterior descending artery, RCA = right coronary artery)

The classification of 91 out of 123 territories (74 %) was identical with CMR and FFR; of the discordant territories, 21 (17 %) were CMR negative and FFR positive and 11 (9 %) CMR positive and FFR negative (See Figs. 2 and 3).

Overall, there was good concordance between the two methods on a per patient basis (k =0.658) and a fair concordance on a per vessel basis (k = 0.433) (See Table 3).

**Table 3:** Per vessel and per patient concordance between CMR and FFR

CMR	FFR result			
	*Per vessel*		*Per patient*	
	>0.75	≤0.75	>0.75	≤0.75
Negative	60	21	7	3
Positive	11	31	2	29

Concordance for the detection of ischemia between CMR perfusion imaging and fractional flow reserve on a per vessel and a per patient basis

Abbreviations: *CMR* cardiovascular magnetic resonance, *FFR* fractional flow reserve

### Concordance between CMR and FFR for various FFR thresholds

Lowering the FFR threshold for FFR improves the percentage agreement for a positive FFR from 56 to 64 % (See Table 4) for result.

**Table 4:** Percentage agreement for different FFR thresholds

FFR threshold		% agreement
0.70	≤0.70	64
	>0.70	80
0.75	≤0.75	60
	>0.75	83
0.80	≤0.80	56
	>0.80	90

Abbreviations: *FFR* fractional flow reserve

## Discussion

Our data shows good concordance between CMR and FFR for the identification of myocardial ischemia in patients with angiographic multi-vessel disease. On a per vessel basis, 91 out of 123 territories demonstrated concordance between the CMR and FFR results (74 %). On a per patient basis there was complete concordance of number and localization of territories in 46 % of patients. However, for the presence of ischemia alone, there is 88 % concordance on a per patient basis.

Despite the high resolution of perfusion CMR, in one third of patients, CMR demonstrated a lower number of ischemic territories than FFR. Agreement was best at the extremes of FFR but less strong for intermediate values.

### The “true” gold standard functional test

Whilst trying to understand the causes of discrepancy between the two tests, it is important to understand that neither of the tests is a true gold standard for ischemia assessment. FFR is recognized to be highly reproducible measure of ischemia [14] but also has a number of limitations. Originally, FFR was validated against a number of non-invasive imaging modalities, with a Bayesian statistical analysis. This involved a combination of all tests as the reference standard and demonstrated a sensitivity of FFR in the identification of reversible ischemia of 88 % with a specificity of 100 % in patients with single-vessel disease [15]. A meta- analysis of FFR vs QCA and non-invasive imaging by Christou et al. demonstrated less favorable results with a sensitivity and specificity of 76 % and 76 % of FFR compared with non-invasive imaging [16]. As such, discrepant results cannot be assigned to one technique or the other, but should be considered as differences.

### Discrepancy between CMR and FFR results

In our study, we demonstrate underestimation by CMR or overestimation by FFR in 33 % of cases. There are four main reasons why two methods measuring the significance of a coronary stenosis may differ:

1. They measure a different pathophysiology and as such have different definitions of a significant coronary stenosis.
2. They use different cut-off values to determine “significance”.
3. A significant stenosis is assigned to a different coronary artery/segment.
4. One of the two tests or both do not measure what they claim to measure.

In the current study each of the four elements contributes to the observed differences.

### Pathophysiology

There are physiological differences in the measures of ischemia by the two tests that may contribute to discrepancies. Stress perfusion CMR indicates altered coronary flow reserve (CFR) assessed by contrast delivery through the entire vasculature of the heart and FFR measures the impact of a coronary stenosis on myocardial perfusion in the territory subtended by that vessel and relies on several assumptions regarding minimal microvascular resistance, which may not be true in all cases.

The influence of the microvasculature is important both as a cause of discrepancy, and also in terms of prognosis. An assessment of this is neglected by FFR, which assumes minimal microvascular resistance, but is incorporated within stress perfusion which assesses the whole vascular compartment. In a recent study [17], patients with intermediate stenoses were assessed by both coronary flow velocity reserve and FFR and patients with a normal FFR but an abnormal coronary flow velocity reserve had a significantly higher major adverse cardiac event rate throughout 10 years of follow-up, regardless of the FFR cut-off applied. Since CMR measures perfusion on a myocardial level, it is plausible that such a discrepancy manifests as a CMR perfusion defect in the presence of a negative FFR.

### Cut-off values

The sensitivity and specificity of a test can be altered by changing the cut-off value used. In the original validation studies the cut-off value for FFR was set at 0.75 [15], although subsequent studies have used a cut-off of 0.80 [18]. The different cut-off values may explain some of the variation of concordance between FFR and noninvasive imaging in the literature. A study by Melikian et al.[10] used 0.8 as the cut-off value and found poor concordance between SPECT and FFR in patients with multi-vessel disease (k = 0.14 on a per patient and k = 0.28 on a per vessel basis). A study by Ragosta et al. [19] used <0.75 as the cut-off value and found better concordance (69 %). In the current study, we demonstrate that reducing the FFR cut-off value results in improved agreement for positive results, while increasing the cut-off value results in improved agreement for negative results. The resulting accuracy is highly dependent on prevalence but the greatest disparity between FFR and CMR occurred between values of 0.7–0.8. A recent meta-analysis by Johnson et al.[20] assessing outcomes in over 9000 lesions evaluated by FFR found the optimal FFR threshold for a composite endpoint of death, MI, and revascularization at 0.67. Interestingly, the FAME 2 data [21] also showed larger benefit for PCI when FFR was <0.65 with a smaller benefit when FFR was >0.65. Whether a lower or higher threshold value is more important for clinical guidance remains unknown for the time being. There is, however, a general tendency towards less revascularization in mild ischemia making a trend towards stricter cut-off values likely.

Similarly, varying the CMR thresholds will result in a variation of concordance with FFR. Our hypothesis was that the higher spatial resolution of perfusion CMR compared to SPECT would result in a higher concordance with FFR due to a better visualization of small perfusion defects. Interestingly, in the majority of discrepant cases in our study, the stenosis with the lowest FFR was identified with both techniques, while less severe FFR results were not seen with perfusion CMR. With SPECT it was suggested [19], that the stenosis with the greatest ischemia is the most evident, leading to visual neglect of subtler perfusion abnormalities. With CMR this is less likely, since perfusion defects usually show as subendocardial defects with normal epicardial perfusion. This allows assessing each coronary artery territory independent of other territories. A recent study by Motwani et al. [22] demonstrated an increase in abnormal territories identified with higher spatial resolution (29 % by standard resolution and 57 % by high resolution imaging (*p* = 0.04)) due to a better visualization of subendocardial defects. However, this may also have been influenced by a higher contrast agent dose used in the high resolution scan. Our study demonstrated concordance on a per vessel level of 74 % which is an improvement on previous studies and may reflect the advantages of higher resolution scanning.

### Variable assignment of perfusion territories

Any study that compares a non-invasive with an invasive technique will be limited by the inability to define exact coronary territories by the 17 segment AHA model. Overlap of segments between the coronary arteries may lead to mis-assignment thus affecting concordance. Additionally, in 2- vessel disease, depending on the functional severity of one stenosis compared with the other, it may be difficult to separate out two small areas of ischemia from one larger more confluent area, again affecting concordance.

The majority of validation studies for both CMR and FFR have been done in a single vessel population, and our data suggests that it is difficult to extrapolate those results to apply to a more complex multivessel population. There are many physiological variables that can affect FFR measurement i.e., presence of scar, collaterals, FFR in small diameter vessels, microvascular dysfunction etc. and these are more likely to be present in patients that have extensive CAD such as our patient population. While in general FFR is normalized for the perfusion area subtended by the interrogated vessel, even a highly positive FFR in a small vessel may only lead to a small amount of myocardial ischemia not detectable by CMR.

### Study limitations

The main limitation of this study is the use of qualitative visual analysis. Quantitative or semi-quantitative perfusion analyses may further improve concordance and accuracy as recently shown in a study comparing visual and semi-quantitative perfusion CMR versus invasive angiography in patients with known or suspected CAD [23]. We have used visual analysis for the identification of perfusion defects as this is more applicable to clinical practice and our goal was the determination of similarities and differences between two clinically used tests.

## Conclusion

This study shows that CMR has good concordance with FFR on a per patient level for the demonstration of ischemia, making it an excellent non-invasive alternative to identify patients suitable for invasive angiography

However, some discrepancies remain in the identification of multiple perfusion defects in patients with multi-vessel disease. There is a general tendency for CMR to shows fewer diseased vessels than FFR. At this stage it is unclear whether CMR underestimates or FFR overestimates the number of ischemic segments in multi-vessel disease, and thus the utility in using CMR to guide revascularization in these patients remains unresolved.

## Abbreviations

AHA, American Heart Association; CABG, coronary artery bypass grafting; CAD, coronary artery disease; CCS, Canadian Class Symptoms; CMR, cardiovascular magnetic resonance; CX, circumflex artery; FFR, fractional flow reserve; HR, heart rate; LAD, left anterior descending artery; LGE, late gadolinium enhancement; LV, left ventricle; MI, Myocardial infarction; PCI, percutaneous intervention; QCA, quantitative coronary angiography; RCA, right coronary artery; RPP, rate pressure product; SBP, systolic blood pressure; SD, standard deviation; SPECT, single photon emission computed tomography
